# Effect of Renamezin upon attenuation of renal function decline in pre-dialysis chronic kidney disease patients: 24-week prospective observational cohort study

**DOI:** 10.1371/journal.pone.0252186

**Published:** 2021-06-07

**Authors:** Hayne Cho Park, AJin Cho, Do Hyoung Kim, Kyu-sang Yun, Juhee Kim, Eun Young Lee, Sang Kyung Jo, So-Young Lee, Kum Hyun Han, Yoon Kyung Chang, Dong-Jin Oh, Young-Ki Lee

**Affiliations:** 1 Department of Internal Medicine, Kangnam Sacred Heart Hospital, Seoul, Korea; 2 Hallym University Kidney Research Institute, Seoul, Korea; 3 Department of Internal Medicine, Soonchunhyang University Cheonan Hospital, Cheonan, Korea; 4 Institute of Tissue Regeneration, College of Medicine, Soonchunhyang University, Cheonan, Korea; 5 Department of Internal Medicine, Korea University Anam Hospital, Seoul, Korea; 6 Department of Internal Medicine, CHA Bundang Medical Center, CHA University, Seongnam, Korea; 7 Department of Internal Medicine Inje University Ilsan Paik Hospital, Goyang, Korea; 8 Department of Internal Medicine, Daejeon St. Mary’s Hospital, The Catholic University of Korea, Daejeon, Korea; 9 Department of Internal Medicine, Myongji Hospital, Hanyang University College of Medicine, Goyang, Korea; University of Alabama at Birmingham, UNITED STATES

## Abstract

Renamezin^®^ is a modified capsule-type oral spherical adsorptive carbon which lowers indoxyl sulfate levels in patients with advanced chronic kidney disease (CKD). This 24-week prospective observational cohort study was performed to evaluate the effect of Renamezin^®^ upon attenuation of renal function decline. A total of 1,149 adult patients with baseline serum creatinine 2.0–5.0 mg/dL were enrolled from 22 tertiary hospital in Korea from April 2016 to September 2018. Among them, a total of 686 patients completed the study and were included in the intention-to-treat analysis. A total of 1,061 patients were included in the safety analysis. The mean age was 63.5 years and male patients were predominant (63.6%). Most of the patients (76.8%) demonstrated high compliance with study drug (6g per day). After 24 week of treatment, serum creatinine was increased from 2.86±0.72 mg/dL to 3.06±1.15 mg/dL (p<0.001), but estimated glomerular filtration rate was not changed significantly during observation period (22.3±6.8 mL/min/1.73m^2^ to 22.1±9.1 mL/min/1.73m^2^, p = 0.243). Patients with age over 65 years old and those under good systolic blood pressure control <130 mmHg were most likely to get benefit from Renamezin^®^ treatment to preserve renal function. A total of 98 (9.2%) patients out of 1,061 safety population experienced 134 adverse events, of which gastrointestinal disorders were the most common. There were no serious treatment-related adverse events. Renamezin^®^ can be used safely to attenuate renal function decline in moderately advanced CKD patients.

## Introduction

Chronic kidney disease (CKD) is the state of chronic functional or structural failure of kidneys which ultimately results in the decline of renal function [1]. The current treatment includes low salt diet, lifestyle modification, blood pressure control and management of underlying disease. However, there are not yet the curative methods to reverse chronic renal damage. Therefore, kidneys ultimately fail to excrete metabolic wastes and the patients undergo renal replacement therapy when uremic symptoms occur.

As renal function declines, various small uremic toxins cannot be effectively excreted through urine. The metabolic wastes from dietary protein such as indoxyl sulfate or p-cresyl sulfate are known to act as uremic toxin which accelerate CKD progression [2]. The AST-120 (Kremezin^®^, Kureha Corporation, Tokyo, Japan), an oral granule-type spherical carbon adsorbent, was developed to delay CKD progression by adsorbing uremic toxins and their precursors and excreting them into feces. Previous observational studies and animal studies demonstrated that treatment with AST-120 reduces the level of indoxyl sulfate [3,4], attenuates renal function decline [5,6] and renal sclerosis [7,8].

Renamezin^®^ is a capsule-type oral carbon adsorbent modified from the AST-120 and was developed by Korean pharmaceutical company (Daewon Corporation, Seoul, South Korea). While Kremezin^®^ uses petroleum pitch as the raw material for spherical carbonaceous adsorbents, Renamezin^®^ is made of furan resin which results in 2 to 10 times greater compression strength [9]. Compared to the previous granule-type Kremezin^®^, it is thought to increase drug compliance and therefore enhance drug absorbing effect. Previous study by Kim et al. demonstrated that daily 6g of Renamezin^®^ effectively reduced the level of indoxyl sulfate as early as 4 weeks after treatment (22.5±13.9% reduction from baseline) [9]. However, there has been no clinical study evaluating the effect of Renamezin^®^ upon attenuation of renal function decline. Therefore, this study was designed to evaluate the efficacy of Renamezin^®^ in attenuating CKD progression among pre-dialysis CKD patients.

## Materials and methods

### Study design and study drug

We performed a 24-week, multi-center, observational study among 22 tertiary hospitals in South Korea. The data were recruited from April 2016 to September 2018. The data within window period of 170±28 days was thought to be acceptable. The study drug was capsule type carbonaceous adsorbent called Renamezin^®^. The patients were recommended to take 7 capsules per time and 3 times a day (6g/day). The amount of study drug was controlled by the investigators based upon side effects and patient tolerability.

### Study population

**Fig 1:**
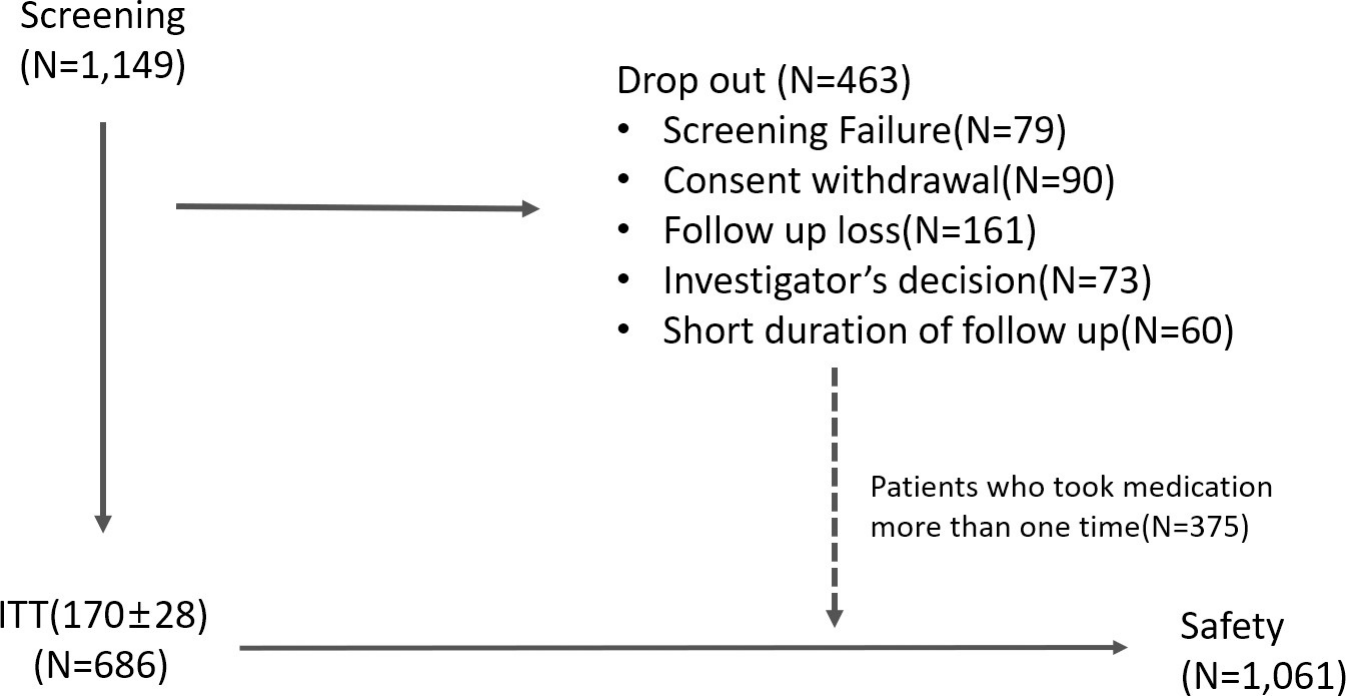
Study population. A total of 1,149 patients were screened for the study and 686 patients who took the study medication for 24 weeks were included in the intention-to-treat analysis. A total of 1,061 patients who took study drug more than once were included in the safety analysis.

A total of 1,149 pre-dialysis adult CKD patients with baseline serum creatinine between 2.0 and 5.0 mg/dL were screened for study enrollment (Fig 1). Among them, a total of 79 patients were failed to be enrolled in the study due to one of the following reasons: those with previous experience of study drug, those with gastrointestinal motility disorder or severe constipation, those with uncontrolled hypertension (systolic blood pressure ≥ 180 mmHg or diastolic blood pressure ≥ 110 mmHg), those with elevated aspartate aminotransferase (AST) or alanine aminotransferase (ALT) over 3 times of upper normal value, chronic alcoholics, those with active infection, pregnant or lactating women, those upon other clinical trials. Another 90 patients withdrew informed consent, 161 patients were lost to follow up, and 60 patients did not reach 24-week goal or had short duration of follow up. Another 73 patients were dropped out due to investigator’s decision including the patients who were inadequate to take medicine due to poor compliance, psychiatric or economic problems. Therefore, a total of 686 CKD patients were included in the intention-to treat (ITT) analysis. A total of 1,061 patients were included in the safety analysis including those who took medication more than once (n = 375).

### Measurement and study outcome

Demographic data were collected at the baseline including age, gender, height and weight, cause and duration of chronic kidney disease, comorbidities and list of medication. Systolic and diastolic blood pressures were measured both at the baseline and the end of the study. All the patients measured serum creatinine and estimated glomerular filtration rate (eGFR) at the baseline and at the end of the study. CKD was defined by eGFR less than 60 mL/min/1.73m^2^. The eGFR was measured using CKD Epidemiology Collaboration (CKD-EPI) equation. The primary outcome was the change in serum creatinine. The secondary outcome was the change in eGFR and the incidence of adverse events during study period. The drug compliance was assessed by an average number of capsules taken by each patient. We divided patients into three groups according to medication compliance: 21 capsules vs. 14~20 capsules vs. 7~14 capsules per day. All the adverse events were collected at the end of the study among safety population.

### Statistical analysis

Statistical analysis was conducted using the SPSS software version 26.0 (SPSS, Inc., Chicago, Ill., USA). For descriptive analysis, data were represented either as mean±standard deviation for continuous variables and frequencies for categorical variables. The student t-test and one-way analysis of variance (ANOVA) were used for comparison. Subgroup analysis was performed to find independent factors related to preservation of renal function. Groups were divided by the change of eGFR (ΔeGFR) during the study, and the group with preserved renal function (ΔeGFR ≥ 0) was compared with the group with decline of renal function (ΔeGFR < 0). Since change in serum creatinine level is not linear over time and can be affected by various factors such as muscle mass and food ingestion, we used eGFR instead of serum creatinine to perform subgroup analysis. Binary logistic regression analysis was performed for comparing subgroups. The forest plot was drawn to find risk factors associated with poor outcome. The P-value < 0.05 was considered statistically significant.

### Ethics statement

The study was approved by the Institutional Review Board Kangnam Sacred Heart Hospital (2016-05-062) and the Institutional Review Board of each participating center: the Institutional Review Boards of CHA Bundang Medical Center, Seoul Eulji Medical Center, Korea University Anam Hospital, Myungji Hospital, Yongin Severance Hospital, Inje University Ilsan Paik Hospital, Sejong General Hospital, G Sam Hospital, Hanyang University Seoul Hospital, Soon Chun Hyang University Cheonan Hospital, Inje University Busan Paik Hospital, The Catholic University of Korea Daejeon St. Mary’s Hospital, CHA Gumi Medical Center, Daegu Catholic University Medical Center, Keimyung University Dongsan Hospital, Chosun University Hospital, Chonnam National University Hospital, Kosin University Hospital, Chung-Buk National University Hospital, Chung-Nam National University Hospital, and Changwon Fatima Hospital. We gathered written informed consent from all the patients before enrollment.

## Results

### Baseline characteristics of the ITT population

A total of 686 patients were included in the ITT analysis. Table 1 shows the baseline demographic and clinical characteristics of the ITT population. The mean age was 63.5 and male was predominant (63.6%). About half of the population had diabetes mellitus and hypertension. Most of the patients had CKD for 1~5 years. The baseline systolic and diastolic blood pressure was 130.9±17.4 and 73.9±11.7 mmHg, respectively. The baseline serum creatinine and eGFR were 2.86±0.72 mg/dL and 22.4±6.8 mL/min/1.73m^2^, respectively. The mean number of pills per day was 19.3, which equates to 5.5g/day. Interestingly, most of the patients (527/686, 76.8%) demonstrated high compliance with the study drug (21 capsules (6g) per day).

**Table 1:** Demographic and baseline clinical characteristics of ITT population.

Variables	Participant (n = 686)
Age (years)	63.5±13.4
Male	436 (63.6%)
Diabetes mellitus	305 (44.5%)
Hypertension	310 (45.3%)
CKD duration	
< 1 year	177 (25.8%)
1~5 years	278 (40.5%)
5~10 years	131 (19.1%)
≥ 10 years	68 (9.9%)
Unknown	32 (4.7%)
Body mass index (kg/m^2^)	24.8±3.6
Systolic blood pressure (mmHg)	130.9±17.4
Diastolic blood pressure (mmHg)	73.9±11.7
Serum creatinine (mg/dL)	2.86±0.72
Estimated GFR (mL/min/1.73m^2^)	22.4±6.8
CKD stages	
Stage 3	115 (16.8%)
Stage 4	472 (68.8%)
Stage 5	99 (14.4%)
Drug compliance	
7~13 capsules/day	36 (5.2%)
14~20 capsules/day	123 (17.9%)
21 capsules/days	527 (76.8%)
Use of ARB or ACEi	403 (58.7%)

ACEi, angiotensin converting enzyme inhibitor; ARB, angiotensin receptor blocker; CKD, chronic kidney disease; GFR, glomerular filtration rate; ITT, intention-to-treat.

### Changes of serum creatinine and eGFR after Renamezin^®^ treatment

**Fig 2:**
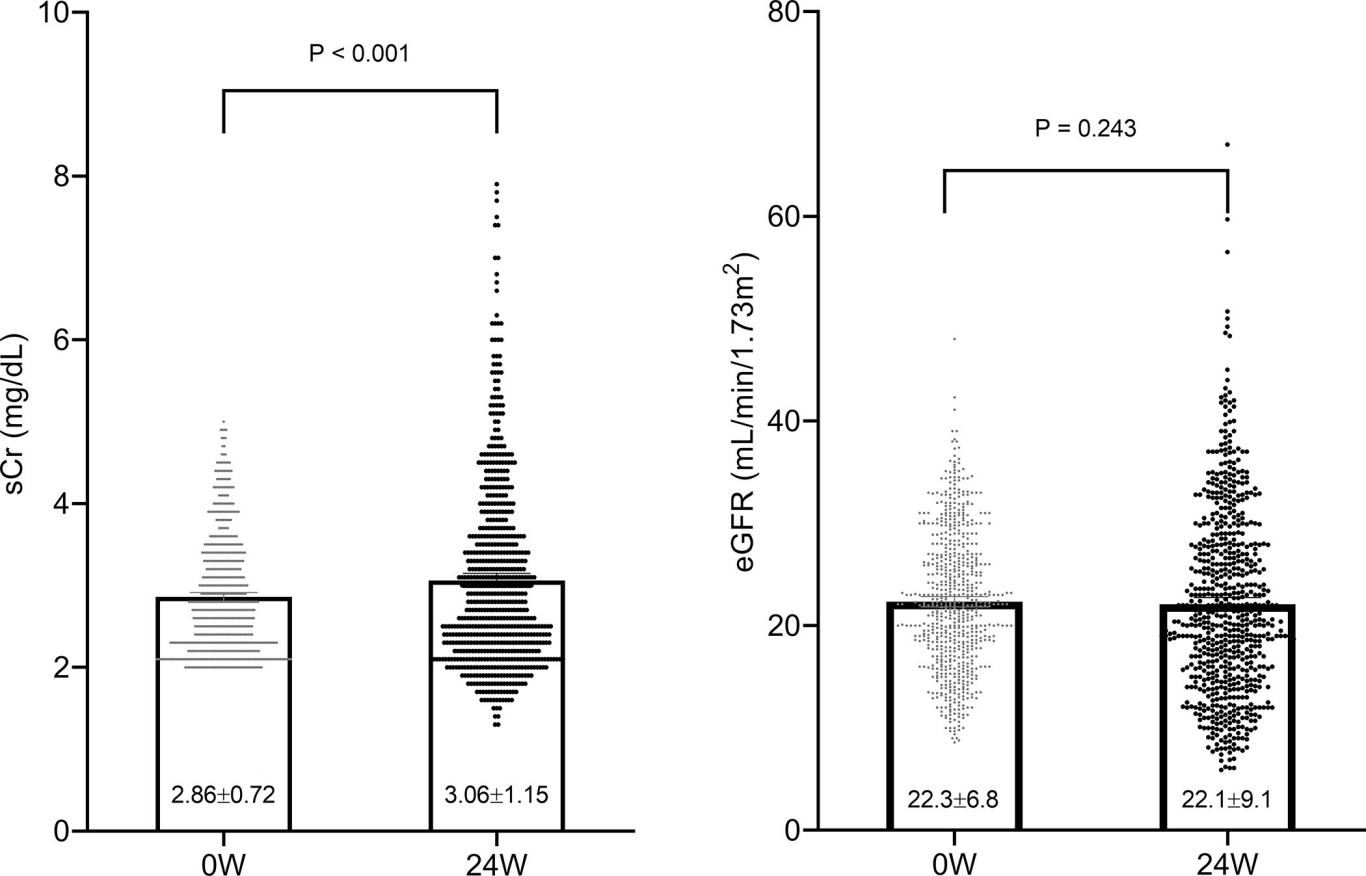
Change in serum creatinine and eGFR after 24 weeks of treatment. (A) Serum creatinine was significantly increased after treatment with Renamezin^®^ (2.86±0.72 and 3.06±1.15 mg/dL, p<0.001). (B) The eGFR did not significantly change after treatment (22.3±6.8 and 22.1±9.1 mL/min.1.73^2^, p = 0.243).

During mean follow-up duration of 173.3±11.1 days, serum creatinine was increased from 2.86±0.72 mg/dL to 3.06±1.15 mg/dL (p<0.001, Fig 2). Therefore, Renamezin^®^ treatment could not prevent the patients from elevation of serum creatinine. However, eGFR did not change significantly over the study period (22.3±6.8 mL/min/1.73m^2^ to 22.1±9.1 mL/min/1.73m^2^, p = 0.243).

### Factors associated with preservation of renal function

To evaluate clinical factors associated with preservation of renal function, we performed binary logistic regression analysis. The patients were grouped according to change of eGFR (ΔeGFR) during study period. The patients with preserved renal function (ΔeGFR ≥0) were compared with those with decreased renal function (ΔeGFR <0) over study period (Table 2). The group with preserved renal function (n = 328) showed older age (65.1±13.0 vs. 62.0±13.7 years old, p = 0.002) and lower proportion of diabetic patients (40.2% vs. 48.3%, p = 0.033). This group also showed lower systolic blood pressure (126.9±16.7 vs. 134.2±17.3 mmHg, p<0.001) and diastolic blood pressure (72.3±11.7 vs. 75.1±11.6 mmHg, p = 0.001) and more narrow pulse pressure (54.7±13.9 vs. 59.1±15.6 mmHg, p<0.001). However, either baseline eGFR (22.7±6.8 vs. 22.0±6.8, p = 0.189) or drug compliance (19.3±3.3 vs. 19.3±3.5 pills/day, p = 0.992) did not differ between groups.

**Table 2:** Factors associated with preservation of renal function.

Parameters	ΔeGFR <0 (n = 358)	ΔeGFR ≥0 (n = 328)	p-value
Age (years)	62.0±13.7	65.1±13.0	0.002
Male (%)	232 (64.8%)	204 (62.2%)	0.478
Diabetes mellitus (%)	173 (48.3%)	132 (40.2%)	0.033
Hypertension (%)	158 (44.3%)	152 (46.3%)	0.584
Body mass index (kg/m^2^)	24.6±3.5	24.8±3.8	0.499
Systolic BP (mmHg)	134.2±17.3	126.9±16.7	<0.001
Diastolic BP (mmHg)	75.1±11.6	72.3±11.7	0.001
Pulse pressure (mmHg)	59.1±15.6	54.7±13.9	<0.001
Baseline serum Cr (mg/dL)	2.95±0.73	2.76±0.68	<0.001
Baseline eGFR (mL/min/1.73m^2^)	22.0±6.8	22.7±6.8	0.189
Number of study pills per day	19.3±3.5	19.3±3.3	0.992
Use of ARB or ACEi	218 (60.9%)	185 (56.4%)	0.233
ΔeGFR	-4.15±3.39	3.99±4.55	<0.001

ACEi, angiotensin converting enzyme inhibitor; ARB, angiotensin receptor blocker; BP, blood pressure; Cr, creatinine; ΔeGFR, absolute change in estimated glomerular filtration rate; eGFR, estimated glomerular filtration rate.

### Patients who can benefit from Renamezin^®^ treatment

**Fig 3:**
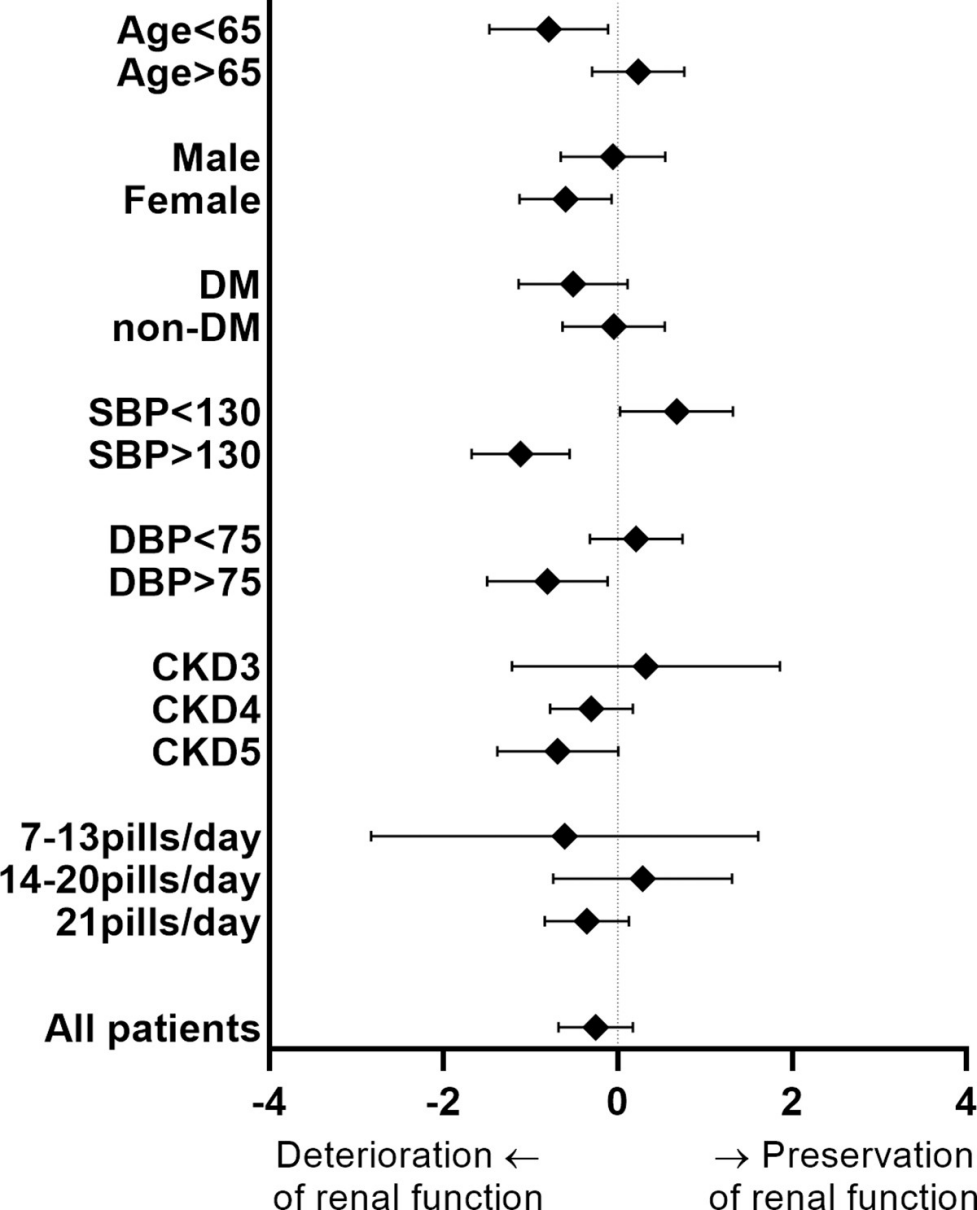
Subgroups who are likely to get benefit from Renamezin^®^ therapy. Elderly patients with age over 65 years old and those with adequate systolic blood pressure control under 130 mmHg were likely to get benefit from Renamezin^®^ therapy. Patient compliance with study drug did not affect renal function preservation in this study. Diabetic patients did not show benefit upon Renamezin^®^ therapy.

To evaluate subgroup who are likely to get benefit from Renamezin^®^ treatment, we performed multivariate logistic regression analysis (Table 3). Elderly patients with age over 65 years old and those with well-controlled systolic blood pressure <130 mmHg were most likely to get benefit from Renamezin^®^ treatment. Since most of the enrolled patients showed good compliance with study drug, there were no statistically significant difference in ΔeGFR according to study pills. Diabetic patients were less likely to get benefit from the study drug. Although statistically not significant, study drug was more likely to be effective in the early stage of CKD (Fig 3).

**Table 3:** Multivariate logistic regression analysis for renal function preservation (ΔeGFR≥0).

Variables	Odds ratio	95% CI	p-value
Age≥65 years	1.505	1.089–2.079	0.013
Male	0.82	0.573–1.174	0.279
Diabetes mellitus	0.752	0.545–1.038	0.083
Systolic BP ≥ 130 mmHg	0.528	0.373–0.747	<0.001
Diastolic BP ≥ 75 mmHg	1.019	0.71–1.462	0.92
Use of ARB or ACEi	0.879	0.637–1.212	0.43
CKD stage			
3	Ref		
4	0.812	0.522–1.263	0.356
5	0.556	0.3–1.03	0.062
Drug compliance			
7~13 capsules/day	Ref		
14~20 capsules/day	1.687	0.773–3.681	0.189
21 capsules/day	1.44	0.709–2.928	0.313

ACEi, angiotensin converting enzyme inhibitor; ARB, angiotensin receptor blocker; CI, confidence interval; CKD, chronic kidney disease; ΔeGFR, absolute change in estimated glomerular filtration rate.

### Adverse events

Adverse events (AEs) in the safety population are summarized in Table 4. During 24 weeks of study period, a total of 98 patients (9.2%) out of 1,061 safety population experienced 134 adverse events. Among them, 38 treatment-related adverse events (3.6%) occurred. The most commonly reported treatment-related adverse events involved gastrointestinal tract including heartburn (5, 0.5%), diarrhea (5, 0.5%), constipation (3, 0.3%), dyspepsia (3, 0.3%), vomiting (2, 0.2%), abdominal discomfort (1, 0.1%), abdominal bloating (1, 0.1%), and nausea (1, 0.1%). One mortality case occurred during the study period, but the exact causal relationship could not be classified.

**Table 4:** Adverse events during the study period.

Description of Adverse events	Safety population (n = 1,061)	
	Events	Patients
Total adverse events	134	98 (9.2%)
Serious adverse events	36	21 (2.0%)
**Treatment-related adverse events**	**39**	**38 (3.6%)**
** • Gastrointestinal disorder**	**28**	**28 (2.6%)**
• Heartburn	5	
• Diarrhea	5	
• Constipation	3	
• Dyspepsia	3	
• Vomiting	2	
• Abdominal discomfort	1	
• Flatulence	1	
• Nausea	1	
• Unclassified	7	
** • Skin disorder**	**3**	**2 (0.2%)**
• Pruritus	1	
• Urticaria	1	
• Rash	1	
** • Edema**	**3**	**3 (0.3%)**
** • Fever or facial flushing**	**2**	**2 (0.2%)**
** • General weakness**	**2**	**2 (0.2%)**
** • Death**	**1**	**1 (0.1%)**

## Discussion

This is the first study to evaluate the effect of novel drug Renamezin^®^ for attenuation of renal function decline in moderately advanced CKD patients. Although Renamezin^®^ did not show significant effect upon delaying renal failure, Renamezin^®^ was beneficial for elderly patients and those with adequate blood pressure control. However, it was not beneficial for those with diabetes mellitus. The treatment-related adverse events were not different from those reported with Kremezin^®^.

Uremic syndrome in advanced CKD involves the accumulation of uremic solutes and toxins in blood which leads to dysfunction of multiple organs and dysbiosis of the gut microbiota [10]. The representative uremic toxin is indoxyl sulfate which is produced from degradation of tryptophan in gut and metabolism by enzymes in liver. The other prototype protein-bound uremic toxin is p-cresyl sulfate which is produced from degradation of tyrosine and phenylalanine in gut [2]. Unlike small water-soluble toxins such as urea or creatinine, protein-bound uremic toxins such as indoxyl sulfate and p-cresyl sulfate are not fully removed by dialysis [11]. Therefore, preventing production of protein-bound uremic toxin from low protein diet and removing them from gut are important to reduce uremic symptoms in advanced CKD.

The AST-120, also known as Kremezin^®^, is the firstly developed oral pharmaceutical agent which consists of spherical carbonaceous adsorbent. It is theoretically beneficial to absorb intestine-origin protein-bound uremic toxins and excrete them to the feces. In fact, Kremezin^®^ effectively lowered the level of indoxyl sulfate [12,13]. However, major randomized controlled trials did not show significant difference in composite of renal outcome (doubling of serum creatinine levels, increase in serum creatinine levels to ≥ 6.0 mg/dL, 50% reduction in estimated glomerular filtration rate (eGFR), initiation of dialysis or kidney transplantation) or all-cause mortality between study and control groups [12,14–16]. One possible reason for negative outcome is that patients find Kremezin^®^ difficult to tolerate because of its unpleasant texture and taste. It is also difficult to take the exact prescribed amount for patients because of granule retention in the oral cavity. Therefore, there has been a need for improving drug compliance.

Renamezin^®^ is a new capsule-type oral spherical carbon adsorbent which can substitute Kremezin^®^. Recent study by Kim et al. showed that Renamezin^®^ can effectively reduce the level of indoxyl sulfate by 20%~30% from the baseline after 4 to 8 weeks of prescription [9]. In addition, Renamezin^®^ can improve patient adherence to medication. Kee et al. demonstrated in their cross-over study that the patient preferred Renamezin^®^ over Kremezin^®^ (65.5% vs. 34.5%) and patient adherence improved in greater degree after switching from Kremezin^®^ to Renamezin^®^ (83.4% to 90.0%, p = 0.057) [17]. However, Renamezin^®^ could not attenuate renal function decline in our 24-week observational study. The reason why the study drug could not delay renal progression is out of the scope of this study. However, there can be several explanations. Firstly, serum creatinine as a primary outcome can be affected by various factors such as muscle mass and food ingestion which can mitigate the change of true renal function. Indeed, previous randomized clinical trials with AST-120 also set primary outcome as either doubling of serum creatinine or developing end-stage renal disease [12,14,15]. However, this study neither was designed as a randomized controlled trial nor was performed for a long duration to evaluate doubling of serum creatinine. Therefore, our study could not prove the effect of study drug in reversing elevation of serum creatinine. Second, we did not compare the rate of renal function decline before and after prescribing medication. Third, there can be a confounding effect of serum albumin and proteinuria of which data were not available in this study. Since indoxyl sulfate and p-cresyl sulfate are protein-bound uremic toxins, the free toxin concentration can be different by the level of serum albumin [2]. Lastly, our study included the patients with more progressed renal function including CKD stage 5 (14.4% of all population). Since the study drug was more effective in the early CKD stage, a large proportion of advanced CKD patients could affect the study result.

Although the study drug did not attenuate renal function decline overall, it was beneficial for some subgroup of patients. The patients with age ≥ 65 years old revealed to get benefit from Renamezin^®^ treatment. The patients who are ≥ 65 years old demonstrated significant preservation of renal function after Renamezin^®^ treatment (OR 1.52, 95% confidence interval 1.102–2.097, p = 0.011). The patients with age ≥ 65 years old revealed to have lower serum creatinine at baseline (2.74±0.67 vs. 2.99±0.74 mg/dL, p<0.001) and lower body mass index (24.3±3.4 vs. 25.2±3.9, p = 0.032) compared to younger patients. Therefore, same amount of study drug may result in better outcome. In addition, the patients with adequate systolic blood pressure control < 130 mmHg were also likely to get benefit from Renamezin^®^ treatment. Finally, the patients in CKD stage 3 were more likely to get benefit from Renamezin^®^ treatment compared to those in more advanced CKD. Our results are similar to those from the previous studies with Kremezin^®^ [18,19]. Previous article suggested that longer prescription of Kremezin^®^ to the patients from the earlier stage of CKD will benefit the most from the therapy [20]. In addition, previous studies with Kremezin^®^ demonstrated that the effect of study drug was larger in the patients with diabetic nephropathy, higher proteinuria, and hematuria [18,19,21,22]. Since carbon absorbent therapy adsorbs uremic toxin, decreases the level of indoxyl sulfate as well as the levels of advanced glycation end products (AGEs), diabetic patients with overt proteinuria will benefit the most from the therapy. However, in our study, the diabetic patients were inferior in treatment effect compared to non-diabetic patients (OR 0.756, 95% confidence interval 0.548–1.043, p = 0.089). Since we neither investigate the status of diabetic nephropathy nor the level of proteinuria but only evaluated the co-existence of diabetes mellitus, our study cannot rule out the effect of Renamezin^®^ upon diabetic nephropathy subgroup. Therefore, further study is warranted to evaluate the effect of Renamezin^®^ upon renal and cardiovascular outcome in the patients with diabetic nephropathy in early CKD stages.

The patients in our study showed high compliance with the study drug. The mean number of pills per day was 19.3, which equates to 5.5g/day. The medication compliance was 92 ± 16.4%, which is higher than that presented in the previous study with Kremezin^®^ (90 ± 11.8%) [12,15]. However, the drug compliance did not affect the renal outcome in our study. This is the opposite finding from the previous study with Kremezin^®^ showing that better drug compliance the better renal outcome. Since most of our patients (76.8%) took the maximum daily dose of Renamezin^®^, the effect of drug compliance upon renal outcome may not be visualized in our study. This should be further elucidated in the future study.

There were fewer adverse events with Renamezin^®^ compared to the studies with Kremezin^®^. A total of 38 (3.6%) patients among 1,061 safety population experienced treatment-related adverse events in our study. In the randomized study with Kremezin^®^ by Akizawa et al. the incidence of treatment-related adverse events was reported as high as 26.2% [14]. The following EPICC-1 and EPICC-2 randomized trial also reported the incidence of adverse events as 18.3% to 21.5% [15]. Together with high drug compliance, lower incidence of adverse events can be a merit of taking Renamezin^®^ as an alternative agent to Kremezin^®^. Recent study by Kee et al. also demonstrated similar medication adherence and efficacy between Kremezin^®^ and Renamezin^®^ while Renamezin^®^ shows lower rates of serious adverse events compared to Kremezin^®^. In addition, the affected tissue or organs in which adverse events occurred were similar to those reported in the previous studies with Kremezin^®^ [12,14,15]. The most common complications were gastrointestinal discomfort including diarrhea, constipation, flatulence, and nausea. Skin related complications were the second most frequent adverse events.

Our study has some strengths. This is the first study to evaluate the effect of novel drug Renamezin^®^ in attenuating renal function decline in a prospective observational cohort study. Although it was not a randomized controlled trial, most of the patients followed up the study schedule and took daily maximum dose of the study drug. On the other hands, our study has some limitations. The study duration was too short to evaluate the effect of the study drug upon renal function decline. We measured serum creatinine and eGFR at only 2 points, at baseline and at the end of the study. Therefore, acute decline of renal function may be mistakenly interpreted as chronic renal progression. In addition, we did not measure important variables such as serum albumin, HbA1c, C-reactive protein, the presence of hematuria, or the amount of proteinuria. At last, we did not measure the level of indoxyl sulfate before and after treatment.

In conclusion, Renamezin^®^ treatment did not slow down renal progression in moderately advanced CKD with serum creatinine 2.0–5.0 mg/dL. However, older patients ≥ 65 years old with adequate control of blood pressure may get benefit from the Renamezin^®^ treatment. Renamezin^®^ may be used safely with high patient compliance as an alternative to Kremezin^®^.

## Supporting information

S1 Data (XLSX)
